# Co-design of an oral health intervention (HABIT) delivered by health visitors for parents of children aged 9–12 months

**DOI:** 10.1186/s12889-022-14174-w

**Published:** 2022-09-24

**Authors:** Jenny Owen, Kara A. Gray-Burrows, Ieva Eskytė, Faye Wray, Amrit Bhatti, Timothy Zoltie, Annalea Staples, Erin Giles, Edwina Lintin, Robert West, Sue Pavitt, Rosemary R. C. McEachan, Zoe Marshman, Peter F. Day

**Affiliations:** 1 School of Dentistry, Faculty of Medicine and Health, University of Leeds, Leeds, LS2 9JT UK; 2 School of Law, The Liberty Building, University of Leeds, Leeds, LS2 9JT UK; 3 Faculty of Medicine and Health, Leeds Institute of Health Sciences, University of Leeds, Leeds, LS2 9JT UK; 4 Bradford District Care NHS Foundation Trust, Children’s Community Services’ (currently on secondment to Better Start Bradford), Bradford, UK; 5 Dental Translational and Clinical Research Unit, School of Dentistry, University of Leeds, Leeds, LS2 9JT UK; 6 Bradford Institute for Health Research, Duckworth Ln, Bradford, BD9 6RJ UK; 7 School of Clinical Dentistry, Faculty of Medicine, Dentistry & Health, University of Sheffield, Sheffield, S10 2TA UK; 8 Bradford Community Dental Service, Bradford District Care NHS Foundation Trust, Bradford, UK

**Keywords:** Dental Caries, Co-design, Behaviour Change, Oral Health, Health Visitors, Parents, Children, Families

## Abstract

**Background:**

Dental caries (tooth decay) in children is a national public health problem with impacts on the child, their family and wider society. Toothbrushing should commence from the eruption of the first primary tooth. Health visitors are a key provider of advice for parents in infancy and are ideally placed to support families to adopt optimal oral health habits. HABIT is a co-designed complex behaviour change intervention to support health visitors’ oral health conversations with parents during the 9–12-month universal developmental home visit.

**Methods:**

A seven stage co-design process was undertaken: (1) Preparatory meetings with healthcare professionals and collation of examples of good practice, (2) Co-design workshops with parents and health visitors, (3) Resource development and expert/peer review, (4) Development of an intervention protocol for health visitors, (5) Early-phase testing of the resources to explore acceptability, feasibility, impact and mechanism of action, (6) Engagement with wider stakeholders and refinement of the HABIT intervention for wider use, (7) Verification, Review and Reflection of Resources.

**Results:**

Following preparatory meetings with stakeholders, interviews and co-design workshops with parents and health visitors, topic areas and messages were developed covering six key themes. The topic areas provided a structure for the oral health conversation and supportive resources in paper-based and digital formats. A five-step protocol was developed with health visitors to guide the oral health conversation during the 9–12 month visit. Following training of health visitors, an early-phase feasibility study was undertaken with preliminary results presented at a dissemination event where feedback for further refinement of the resources and training was gathered. The findings, feedback and verification have led to further refinements to optimise quality, accessibility, fidelity and behaviour change theory.

**Conclusion:**

The co-design methods ensured the oral health conversation and supporting resources used during the 9–12 month visit incorporated the opinions of families and Health Visitors as well as other key stakeholders throughout the development process. This paper provides key learning and a framework that can be applied to other healthcare settings. The structured pragmatic approach ensured that the intervention was evidence-based, acceptable and feasible for the required context.

**Trial registration:**

ISRCTN55332414, Registration Date 11/11/2021.

**Supplementary Information:**

The online version contains supplementary material available at 10.1186/s12889-022-14174-w.

## Background

Dental caries (tooth decay) in children is a national public health problem [1], with a quarter of five-year-old children in England experiencing tooth decay [2]. Caries has numerous negative consequences for children including pain, infection, difficulties eating and sleeping and reducing quality of life [2–5]. Dental caries and its management remains the leading reason for hospital admission in England for 5 to 9-year olds, with 25,702 admissions in 2018–2019 [6]. Moreover, there are significant health inequalities with children from deprived areas at increased risk of poor oral health [7].

### Early intervention and oral health

National guidelines identify strong evidence for the effectiveness of twice-daily parental supervised toothbrushing with fluoride toothpaste and limiting sugary foods and drinks [8]. There is, however, a clear research gap in how to empower parents to undertake optimal oral health behaviours at home. Toothbrushing should commence on eruption of the first primary tooth (between six and twelve months old) with parents brushing or actively assisting with brushing up to the age of seven years old using the appropriate amount and strength of fluoride toothpaste [8–11]. For this paper we will abbreviate these toothbrushing behaviours to parental supervised toothbrushing (PSB). As toothbrushing is a habitual behaviour, when initiated in early childhood, it is more likely to be sustained and lead to long term oral health in adulthood [12, 13]. Public Health England (PHE) has advised how the wider early years workforce, such as health visitors, could be trained and supported to provide oral health advice to families, thus supporting the ‘Making Every Contact Count’ (MECC) approach [14].

### Health visitors

Health visitors are a key provider of support and advice for parents of young children (0–5 years). According to NHS Workforce Statistics, in 2020 there were 6,672 full time equivalent health visitors working in the NHS in England [15]. Health visitors are registered nurses and /or midwives who have the additional university qualification of Specialist Community Public Health Nurse. They often lead teams, with a mixed range of skills, and provide an evidence based public health service to children and families, groups and communities. Health visitors aim to enhance health and reduce health inequalities through a proactive, universal service for all children 0–5 years old. Health visitors therefore have an ideal opportunity to support families to adopt optimal oral health habits (including PSB and limiting sugary foods and drinks). The universal visits undertaken by health visitors with parents of children aged 9–12 months are one such opportunity [16, 17]. One of the five core mandated visits, conveniently, this visit is timely owing to the recent eruption of the primary dentition. National guidance [11, 18] advocates the inclusion of an oral health conversation at this visit, however, there is limited evidence of the effectiveness of such conversations [19]. Our research [20] and that of others [21, 22] has identified several barriers that limit the opportunity, consistency and effectiveness of these conversations. Health visitor training in oral health can vary significantly between localities and is predominantly provided as an online training resource. Prior to the publication of the ‘Best start in life and beyond: Improving public health outcomes for children, young people and families’ guidance document in 2021, [23] annual oral health sessions were offered to all health visitors and supported by resources. The 0–19 service specification for oral health indicated that training should be mandated annually via the health visitors’ online education platform.

### Complex behaviour change interventions and co-design

PSB is a complex behaviour with many individual and external determinants, which require a multileveled approach addressing the varying barriers to performance. There are a number of approaches to developing complex interventions, including the widely cited complex intervention development framework from the Medical Research Council (MRC) [24]. Much attention has been paid to increasing the use of behaviour change theory within intervention development [25–27]. Recognising and harnessing the expertise of key stakeholders in the development of complex interventions is essential [28, 29]. Participatory research designs involve the active participation of stakeholders in service or intervention design [28, 30]. Terminology associated with participatory research designs is often used interchangeably [31] and has been criticised for being poorly defined [32]. For the purpose of this study, co-design is defined as a process by which stakeholders are involved in the design of an intervention to ensure that the result is usable and meets their needs [33].

This paper will outline how the intervention was developed by a multidisciplinary research team using principles of co-design, including the development of intervention resources [34]. This intervention has been tested as part of an early-phase feasibility study [35] and is known as the Health visitors delivering Advice in Britain on Infant Toothbrushing (HABIT) intervention. This paper focuses on the iterative and comprehensive co-design element of the project by describing the journey from initial development through to the findings of the feasibility study. We discuss how the findings were incorporated into the study design to ensure the intervention was feasible and deliverable within practice and the research setting. Other papers have focussed on individual aspects of the co-design process, for example the organisational barriers to oral health conversations [20], the feasibility study including the design [35] and mixed methods evaluation [36, 37].

## Methods

Throughout the paper, “health visitors” will be used as a collective term representing health visitors and nursery nurses who took part in the HABIT intervention.

### Underpinning theoretical framework and generic intervention development

The HABIT intervention was developed using an intervention mapping approach producing a theoretically informed and evidence-based intervention that included extensive community and stakeholder engagement, and a robust needs assessment [38]. This approach followed MRC complex intervention development guidance [39], and included undertaking systematic reviews [40, 41] and qualitative interviews with local populations in Yorkshire to describe the barriers to PSB [42]. Each step of this process was underpinned by the Theoretical Domains Framework (TDF), a framework of the key determinants of behaviour developed from several behaviour change theories, [43, 44] and behaviour change taxonomy [27].

### Stages of intervention development

**Fig. 1:**
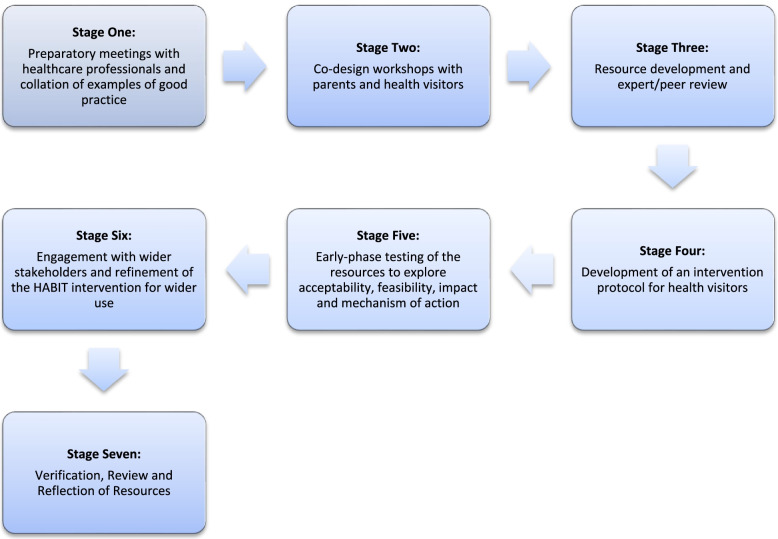
Diagram to show the stages of the co-design intervention development process

Figure 1 shows the seven stage co-design process that was undertaken. The process involved: (1) Preparatory meetings with healthcare professionals and collation of examples of good practice; (2) Co-design workshops with parents and Health Visitors; (3) Resource development and expert/peer review; (4) Development of an intervention protocol for health visitors; (5) Early-phase testing of the resources to explore acceptability, feasibility, impact and mechanism of action; (6) Engagement with wider stakeholders and refinement of the HABIT intervention for wider use, (7) Verification, Review and Reflection of Resources. These stages of development have been mapped to the GUIDED checklist and reporting guidance [45] which provides a structured approach for reporting intervention development (See Additional File 1).

Stages one to four were completed over a period of approximately six months. Stage Five took approximately 18 months to carry out the early-phase feasibility study, and stages six and seven were conducted over approximately 12 months.

Ethical review and research governance approvals were obtained for different stages of the co-design process [stage two (160517/PD/229), stage five (17/YH/0301) and stage six (180620/PD/301)], Health Research Authority (IRAS ID 230315) and NIHR CRN Portfolio Adoption.

### Stage one: preparatory meetings with healthcare professionals and collation of examples of good practice

Using professional contacts, eight face-to-face and telephone meetings were arranged with 18 stakeholders from across England to collect existing resources used by health visitors as part of conversations about oral health during mandatory home visits. The meetings provided an opportunity to gain a deeper understanding of current practice, the suitability of existing resources, and to identify what further resources may be needed to support oral health conversations between health visitors and parents. Resources collected included leaflets, toothbrushes, toothpaste, vignettes and mouth models. Where meetings could not be arranged, resources were requested to be sent via email or post. All resources were anonymised, as far as possible, so that neither their origin nor stakeholders’ identity or organisation were identifiable.

### Stage two: co-design workshops with parents and health visitors

The anonymised resources were used as the basis for discussion in co-design workshops. Separate workshops were held with parents of children aged 9–12 months and Health Visitors. Parents were recruited via children’s centres or nurseries in West Yorkshire, UK. The children’s centres and nurseries were purposively sampled to maximise demographic diversity. Parents were approached in a variety of ways, including poster advertisements or through written invitation by the centres/nurseries on behalf of the research team. Health visitors in West Yorkshire were approached to take part via an email invitation, accompanied by an information sheet and asked to contact the research team directly if interested in participating.

During the workshops health visitors were asked to reflect on their own practice and the oral health resources they currently or previously had access to/used. The topic guides for both parent and health visitors (see Additional Files 2 and 3) were mapped onto the Theoretical Domains Framework (TDF) [43, 44], with questions addressing 14 different domains including knowledge, skills, social influence, memory and emotions. Health visitors and parents were both asked to explore the anonymised resources and express their opinions on; 1) the strengths and weaknesses of the different resources; 2) how the resources could be used in supporting parents to adopt evidence-based optimal oral health habits. Both groups were then asked to identify their favourite top three resources and the reasons behind their choice. They were also invited to suggest ‘something else’ that they felt would be beneficial to add to them that was not already contained within the existing resources. Individual interviews were undertaken with parents and health visitors who were unable to attend the workshops, but who still wished to participate in the study. Parents and health visitors who took part in individual interviews were asked to explore the resources in the same way as those who participated in the workshops, although the interview format enabled the reasoning behind their comments to be explored in greater depth. Workshops and interviews were audio recorded and transcribed. The transcripts were analysed using a combination of inductive and deductive approaches based on the TDF [43, 44] and thematic analysis [46] using NVivo 10.

### Stage three: resource development and expert / peer review

The research team used feedback from stages one and two in conjunction with behaviour change theory [43, 44] to design the resources to be used as part of the intervention. The transcripts from the interviews and workshops were analysed to explore the potential barriers to parents developing and maintaining optimal home-based oral health behaviours for their child, and for health visitors in delivering supportive oral health advice. Resources were developed in a range of formats to facilitate behaviour change by addressing the potential barriers highlighted by health visitors and parents.

The resources were then subject to expert peer review by colleagues from Public Health England (PHE) to ensure they complied with the current national oral health guidance[47].

### Stage four: development of an intervention protocol for health visitors

Health visitors were invited to take part in the early-phase feasibility study [35]. As part of a wider oral health training day health visitors familiarised themselves with the HABIT intervention and resources developed in stages one-three. Discussions were held with the health visitors on how best to structure the oral health conversation during the 9–12 month visit. To capture further feedback and review consistency of intervention delivery, a diary was developed and discussed in which the health visitors could reflect on each oral health conversation with parents.

### Stage five: early-phase testing of the resources to explore acceptability, feasibility, impact and mechanism of action

The HABIT intervention and resources were subjected to an early-phase evaluation to explore acceptability, feasibility, impact and mechanism of action [35]. A mixed methods evaluation was undertaken and is reported in detail in other publications [36, 37]**.**

### Stage six: engagement with wider stakeholders and refinement of the HABIT intervention for wider use

A dissemination event was organised to present the findings from the early-phase evaluation undertaken in Stage Five. A wide range of stakeholders and study participants were invited. The research team presented the findings and showcased the HABIT intervention and resources. The delegates were divided into groups, asked to review two of the resources and provide feedback relating to what aspects they liked, what they would improve and what they would lose or remove. In the final session of the day a structured conversation was facilitated where delegates could provide feedback on the preliminary results, identify remaining unknowns and explore the next steps.

### Stage seven: verification, Review and Reflection of Resources

Following the dissemination event, the research team reflected on the feedback provided. A review of the resources was undertaken by two independent researchers who were not involved in the original intervention or resource development. This involved the comparison of oral health information provided to the current Public Health England guidance [47] and to verify the resources to ensure they targeted the behaviours originally intended using the Theoretical Domains Framework (TDF) [43, 44, 48].

## Results

### Stage one: preparatory meetings with healthcare professionals and collation of examples of good practice

A pool of 18 stakeholders and health care professionals were approached to provide the resources they use at the 9–12 month visits. The research team delivered a one-day training event (which was delivered on two separate occasions), for health visitors where they introduced the HABIT project, discussed the need for resources, and collected attendees’ feedback on what type of support or aids would facilitate their day-to-day practice. In addition, individual meetings with health visitors, senior dental public health managers, members of oral health promotion teams, senior oral health improvement practitioners and health improvement facilitators were arranged to discuss their perspectives and the resources they use.

Over the course of two months, nine resource sets were collected from eight services across England. Some of the stakeholders reported absence or limited availability of oral health resources used at the 9–12 month universal visits. Instead, they shared resources that are used at some point between conception and child age of 30 months, therefore they were using more generic material and not using resources that were specifically targeted for the 9–12 month age range.

The shared resources fell broadly under two categories: those for use in a group setting and those for use on a one-to-one basis. With regard to group-facing resources, these were provided by one service that is geographically located in areas with high levels of dental disease in children. The team who shared this resource set noted that due to financial cuts oral health training for groups of parents had been cancelled, thus the resource set in question has not been used for some time. Nevertheless, they emphasised that health visitors and parents used to find it particularly helpful for facilitating oral health conversations and introducing optimal oral health behaviours. With regard to the one-to-one resources, these varied from a single A5 leaflet to models of teeth for toothbrushing demonstration. The majority of stakeholders noted that they shape oral health conversations around leaflets and items related to optimal oral health (conversations around free-flow cups, for example). Others, however, reported that in recent years the availability of resources has decreased significantly. Consequently, those who had no resources reported borrowing publicly available resources used in neighbouring localities. Others noted that a toothbrush and toothpaste were the only resources they had (used for demonstration only and were not given to parents). Despite the difference in resource availability, all stakeholders emphasised the value of appropriate oral health resources in encouraging parents to brush their child’s teeth, thereby optimising oral health behaviours.

### Stage two: co-design workshops with parents and health visitors

A total of three workshops with 14 parents of children aged 9–12 months, and a total of three workshops with 15 health visitors were undertaken. An additional six interviews were conducted with three parents and three health visitors. Interviews and workshops took place between June and October 2017.

#### Findings from parent workshops and interviews

The information and support provided to parents was varied, with many parents stating that they had received little information about oral health directly from their health visitor. Several parents, however, suggested that they would have liked more advice at the time, with others stating that they felt they needed support sometime after the visit as their child grew older. Parents obtained oral health information from a variety of sources, including online websites, peers and family members; and only rarely was advice sought or received from dental professionals. Difficulty finding a dentist to register with in the local area was often cited as a barrier to obtaining information about oral health for their child. Participants agreed that information about oral health would be useful, particularly for first time parents, around the time that teeth first erupt.

Feedback upon the existing written resources collected in Stage One was that improvements could be made. Parents highlighted the importance of being provided with key messages that were easy to read, without too much detail or too many pictures, which distracted from the content.

“For me it’s, this is just all a bit too stimulating and there’s just too much going on. You know, there’s stuff to read everywhere and pictures everywhere.” (Parent)

Priorities for the content of key messages included what age to begin brushing, information on when parents should first attend the dentist with their child, weaning support and healthy eating advice. Parents also highlighted the need for practical information about how to brush.

“When they should start doing it, what you should use, what’s the best thing to use, how to do it. Step-by-step.” (Parent)

Some parents identified how their child’s challenging behaviours may become a barrier to PSB and suggestions around how to overcome these behaviours was important.

“And…yeah some acknowledgement that it may not be straightforward then I think that, I think that would be helpful actually” (Parent)

Parents varied in their preferences about how resources on oral health should be provided; some preferred for the information to be written, in the form of a leaflet, and others stated a preference for electronic resources, such as websites or videos. However, a consistent theme was the need for all the necessary information to be presented in one place, in a concise fashion. Many parents identified the importance of the oral health discussion with the health visitor, which would encourage them to engage with the resources and or retain for future use:

“I think it’s better a person telling you rather than a leaflet telling you” (Parent)

The way in which information was delivered was particularly important to some parents who prioritised the importance of having an open, non-judgemental conversations with their health visitor:

“And [health visitor] tells me in a very sort of patronising way, you know, the way that she presents information… And that, that really has put me on the defensive.” (Parent)

Parent’s experience of conversations with health visitors appeared to contribute significantly to their perceptions of the usefulness of the service and information provided by health visitors. Experiences varied significantly with some parents viewing the contacts as more of a ‘tick box’ exercise and others placing significant value upon the support and information received from their health visitor.

#### Findings from health visitor workshops and interviews

Health visitors who participated in the interviews and workshops highlighted the high levels of tooth decay experienced by children in their local area. They perceived facilitating good oral health to be an important aspect of their role. Often their conversations were initiated by giving out a toothbrushing pack (consisting generally of toothbrush and toothpaste). There was variation in the level of detail given to families about oral health and health visitors described how information may be prioritised depending upon the particular circumstances of each family. For example, the discussion may focus more on healthy eating if the health visitor noticed that the child was being given sugary foods or drinks.

One difficulty identified by the health visitors was the number of topics which needed to be covered within the 9–12 month visit. Some felt that they did not have enough time to cover the topic of oral health in detail:

“…we don’t really focus on it. You know, we touch on it, ‘are you registered, you know? You need to brush their teeth, you need to use this much toothpaste on a soft brush’ and, you know, that’s pretty much it, you know. It is a bit of a sort of whistle stop…” (Health visitor)

“So if, if a parent comes in with a specific problem, it might be about sleep or something, you do devote an awful lot of time to that. And then other things, it’s kind of a quick mention. So I think that, that’s a real difficulty isn’t it.” (Health visitor)

Information about oral health was predominantly delivered verbally. Health visitors described the limited availability of resources to support conversations about oral health:

“So I don’t know whether it’d be a leaflet or, or something. We don’t sort of have anything like that for them to sort of keep or to refer back to…” (Health visitor)

Feedback was obtained on the resources collected during Stage One. Health visitors valued resources which were visually engaging (i.e., bright, colourful) and those with pictorial representations. They felt that many of the existing resources were overcrowded with text and/or pictorial information and stated a preference for the resources to contain ‘key’ information only, in a ‘bullet point’ style. The size of the resources was also important; many health visitors suggested that resources should not be heavy or bulky for them to carry and suggested that ‘pocket size’ would be ideal.

“It needs to be small as well cause we all carry heavy bags don’t we.” (Health visitor)

The health visitors identified that personal preference was likely to play a role in families’ attitudes about different resources. For example, some may prefer written information in the form of a leaflet and others may prefer electronic resources (such as a website or videos). The availability of resources in different formats was also perceived to promote accessibility, for example; some parents may be unable to read a written leaflet but may be able to access or prefer video resources.

“Cause I don’t, as you were saying, leaflets don’t always work for parents. They think oh yeah, yeah, oh, another leaflet. It’ll just go in the bin.” (Health visitor)

“And, you know, if there is a good website that you can signpost to them I’m more than happy to do it, you know…” (Health visitor)

Some of the health visitors, especially those who work with families living in the most deprived areas, mentioned that some parents would not be able to access the online resources and thus would be denied an opportunity to learn how to ensure their child’s oral health.

One suggestion made was that a set of model teeth might be useful on which to demonstrate the action of toothbrushing:

“…we could have a little, a little teeth with their brush and show them how to do it.” (Health visitor)

### Stage three: resource development and expert / peer review

- Resource Development

As discussed, the parents felt that a supportive conversation with health visitors was the most important part of the oral health component of their visit. This conversation should be accompanied by appropriate resources to supplement the discussions.

Informed by the preceding research work, and findings from the workshops and interviews, six broad topic areas were identified to form the basis of the HABIT resources. The topic areas are listed below, with a brief explanation of the key message/s.

1. No Second Chance—(Why oral health is important and consequences of dental decay)
2. Toothbrushing Knowledge (Toothbrushing advice, e.g., twice daily with a fluoride toothpaste, strength and amount of fluoride toothpaste to use and parental supervised brushing until at least the age of seven)
3. Toothbrushing Skills (Support and tips for brushing children’s teeth, e.g., positioning options and techniques for effectively brushing a child’s teeth, systematic approach and brushing all surfaces of the teeth)
4. Managing Behaviour (Providing reassurance that brushing children’s teeth is often challenging and providing tips to make brushing easier)
5. Diet Knowledge (Information around healthy food, drinks and snacks, frequency of sugar, advice to only drink milk and water and use of a free flow cup over 6 months of age)
6. Social Influence (Empowering parents to work with other family members involved in their child’s care around the importance of brushing children’s teeth)

The key messages provided in the resources were informed by Stage One and Two of the project and the previous programme of research, which highlighted the individual, social and structural factors that influence PSB [38, 40–42]. The key messages aimed to facilitate behaviour change by targeting the potential barriers to PSB including knowledge, skills, self-efficacy, routine setting and behavioural regulation.

The key messages provided a structure for the supportive materials and resources which were designed in two formats; a leaflet and a website. First, a fold up, pocket-sized leaflet, which contained short sentences of essential information on each of the six key topic areas. At the back of the leaflet was an action plan, which consisted of a list of positive oral health behaviours, e.g., ‘Brush my baby’s teeth twice a day with a fluoride toothpaste’ and ‘Avoid sugary foods and drinks an hour before bedtime’. The action plan was provided to aid behaviour change as parents could choose one or two key areas to focus on, and in conjunction with the health visitor, discuss how to achieve this goal. Based upon feedback from Stage Two of the project, text was kept to a minimum and the leaflet was designed to be colourful (a different colour associated with each key message) and engaging (one simple illustrative picture per message).

Second, a website housing short two-to-five-minute video vignettes on each of the six key message topics was developed. The video vignettes included key messages from Public Health England’s ‘Delivering Better Oral Health’ toolkit [47], demonstrations, practical examples and tips, as well as parents sharing their own stories, challenges and solutions. These stories include parents from different backgrounds to maximise their appeal and engagement with different parent groups. The involvement of parents within the video came from earlier community engagement work. These peer stories, which other parents could relate to were identified by local communities as being far more powerful than messages from a dental professional. The website was designed to coordinate with the leaflet and the colours and pictures associated with each key message were consistent on each. The address for the website was also printed on the front of the leaflet to encourage parents to visit the website and for health visitors to promote it as a trusted source of information.

b) Expert/Peer review

HABIT resources were reviewed by a Consultant in Public Dental Health, who was also the National Lead for Oral Health Improvement and two Senior Dental Public Health Managers, all employed by Public Health England. These colleagues provided national leadership to the area of oral health promotion and were responsible for writing and updating the national oral health guidelines [47]. Their detailed feedback was to ensure that key messages aligned with their published materials. The resources were also reviewed by a group of 25 healthcare colleagues from the 0–19 Healthy Child Programme in Yorkshire and the Humber. The comments received from both groups focused on (i) subtle changes in language; (ii) providing positive examples such as multiple clips of different parents brushing their child’s teeth, use of a two toothbrush technique so that the child has something to hold while their teeth are being brushed, squirting out food pouches into a bowl and providing examples of healthier snacks for teeth; (iii) explanations around what is a free flow cup and at what age these should be used from and when the use of a bottle should be stopped; and (iv) ensuring the HABIT resources aligned with wider public health activities such as Dental Check by One and providing captions aligning to key messages such as “squashes and fizzy drinks have no place in children’s diets”.

### Stage four: development of an intervention protocol for health visitors

Eight health visitors attended a training day on the HABIT intervention. The health visitors watched a series of novel television-based programmes developed by “SOAP” designed to support early-years professionals’ oral health knowledge (www.soap.media). These innovative resources had been reviewed by Public Health England to ensure they were compliant with current national guidance [47]. The programmes focused on different age groups (0–2 year olds, 2 year olds, 3–4 year olds), and discussed with a panel of health experts and parents key issues pertinent to each age group. After viewing each programme, in small groups, the health visitors reflected on and discussed what they had seen. Moreover, they had a chance to discuss any questions they had with a dental hygienist and therapist, and a paediatric dentist from the research team.

The health visitors then viewed the HABIT resources and videos, providing an opportunity to discuss the resources and their implementation. The health visitors worked with the research team to agree upon a delivery protocol (a standard format) on how the HABIT intervention would be delivered. Health visitors raised key features they wanted included within the protocol including: the importance of the initial oral health conversation; a visual hands-on demonstration of toothbrushing technique, either with the child or on a plastic set of teeth; and for the conversation to identify and focus on the oral health issues, which were most important to parents. A simple, five stage protocol was finalised to guide delivery of the oral health conversation during the 9–12 month visit. This included;

- 1) Handing out the dental pack consisting of a toothbrush, toothpaste and HABIT leaflet, and starting the conversation about toothbrushing.
- 2) Asking parents to brush their baby’s teeth and then provide a demonstration of toothbrushing technique (using a set of plastic model teeth if a hands-on demonstration wasn’t possible due to lack of cooperation from the child).
- 3) Identifying and discussing the most important issue to parents regarding oral health and supporting patents to identify their own solutions to overcome challenges faced.
- 4) Signposting to the leaflet, website and videos, using these to guide and support the conversation between health visitors and parents.
- 5) Encouraging parents to create an action plan and recording how they intend to implement their plan over the next two weeks. The action plan was written on the HABIT leaflet, which contained suggestions of areas parents may wish to focus on. These included: ‘using a smear of fluoride toothpaste’, ‘stick to milk and water to drink’, ‘make toothbrushing as fun as possible’. However, space was provided to allow parents to create unique goals should they so wish.

The one-day training session aimed to ensure all health visitors delivering the HABIT intervention had up-to-date oral health knowledge in line with national guidelines [47] and all had participated and agreed on how it would be delivered. The discussions during the day led to the finalised protocol to guide the delivery of the HABIT conversation between health visitors and families. A structured diary was finalised as a method of recording how the visit went, the consistency of intervention delivery, what resources were used and provided an opportunity to reflect on their conversations after each visit. Additional File 4 provides the TiDieR checklist outline the HABIT intervention as delivered throughout the feasibility study.

### Stage five: early-phase testing of the resources to explore acceptability, feasibility, impact and mechanism of action

Detailed findings from the HABIT early-phase feasibility study are beyond the scope of this paper and are reported in separate publications [35–37]. In summary, the feasibility study identified that the HABIT intervention was acceptable to parents, feasible for health visitors to deliver and provided a strong signal of improved PSB behaviours at three months after the intervention. Parents felt their health visitors were trusted people from whom they were happy to receive the intervention. The parents felt that the intervention provided them with the support and encouragement to know that they were doing the right thing, e.g., starting to brush their baby’s teeth on eruption of the first tooth. Both health visitors and parents highlighted how important the timing of advice provision was and health visitors discussed that oral health information integrated well into their existing conversations about health promotion. A number of refinements were identified which are discussed together with findings from stage six.

### Stage six: engagement with wider stakeholders and refinement of the HABIT intervention for wider use

Following the completion of the early-phase feasibility study, preliminary results were presented at a dissemination event. Sixty-six delegates attended, including some of the health visitors who had delivered the HABIT intervention as well as many other health and early-years professionals including representatives from: Bradford District Care NHS Foundation Trust Research, Health Visiting and Dental teams, Public Health England, Bradford Local Authority, Born in Bradford/Better Start Bradford, Oral Health Promotion Group, British Society of Paediatric Dentistry and University of Leeds.

As part of the dissemination day, the delegates reviewed the HABIT resources in small groups and provided valuable additional feedback in the form of what they would ‘Keep’, ‘Improve’ and ‘Lose’. There were very few comments relating to aspects that people wanted to ‘Lose’ from the resources, however, there were various elements that were liked and several areas where improvements could be made. Feedback included; improvements to format of the leaflet, increased font size, or highlighting particular information to be more prominent. The videos were well received, with some delegates commenting that they felt true to life with good examples of parenting tips or safer snacks. Some delegates provided very constructive feedback suggesting improvements to the clarity of certain aspects of advice, as some visual elements could be misunderstood without the supportive audio. For example, when foods and drinks that are not safe for teeth are shown in the video, without the supportive audio, these could be seen as acceptable for children to consume as they are in an oral health video.

The event also enabled delegates to review the results and discuss how to take the HABIT intervention forward. Comments provided on the day included: widening the accessibility of the videos, such as translating the resources into other languages and the use of subtitles on the videos, suggestions for less written text and more visuals to support the language barrier concerns; there were requests for additional links from the website to other useful resources and some requests for the inclusion of more toothbrushing demonstrations to highlight the correct techniques.

There were two main areas identified as needing further development before progressing to a definitive study or trial:

1. HABIT resources:
  updating of the consent of parents and their children to continue to appear in the HABIT videos;
  working with key local communities, with high levels of early-childhood decay, to ensure the videos and resources were appropriate, for example, if English was not a first language and to comply with other accessibility guidance [49]; and
  address the utility of the HABIT intervention to enable them to support different universal mandatory home visits that health visitors undertake for children aged 0–30 months.
2. Health visitor training – feedback from the dissemination day and the qualitative interviews with health visitors and parents identified inconsistencies in the delivery of the HABIT intervention [36, 37]. Refinements to the HABIT training include preparation work for the delegates before they attended, such as watching the online SOAP resources, HABIT videos and videos showcasing examples of “effective” HABIT conversations. This provided additional time during the training for health visitors to practice the structure of the “HABIT” oral health conversation using forum theatre, a type of role-play involving actors and reinforce the importance of signposting parents to the online HABIT resources and the use of the action plans. Furthermore, the training would provide further opportunities to work with health visitors to identify how best to monitor the fidelity of these conservations.

### Stage seven: verification, review and reflection of resources

The HABIT videos, leaflet and website were reviewed and verified against the Theoretical Domains Framework (TDF) [43, 44] and Delivering Better Oral Health [8] guidance. Each resource was independently coded by two researchers who then subsequently met, reviewed their coding, and agreed on any dissimilarities [48]. The findings of this exercise showed that all 12 of the TDF domains were addressed across the HABIT resources which aligns with the findings from the initial work to identify the barriers to PSB [40–42]. Similarly, all oral health messages were consistent with Delivering Better Oral Health guidance, and the majority of guidance points were covered with the exception of breast feeding and the application of fluoride varnish at dental appointments (see Additional File 5 for a copy of the summary table of the mapped domains).

## Discussion

This paper describes the co-design of HABIT, an oral health intervention to be used by health visitors at the 9–12 month developmental review visit. The co-design methods ensured the oral health conversation and supporting resources incorporated the opinions of families and health visitors as well as other key stakeholders at multiple points along the developmental pathway. Review of the final resources by mapping the content to the Theoretical Domains Framework [43, 44] and Delivering Better Oral Health [47] an approach identified by previous research [48], allowed the team to assess the quality of the oral health information provided and ensure it aligned to current guidance, as well as assess if the intervention did indeed target the barriers to optimal oral health practices. The co-design approach to development ensured that the HABIT intervention was acceptable to parents, feasible for health visitors to deliver and provided a strong signal of improved PSB behaviours at three months after the intervention, as shown by the findings from the feasibility studies [36, 37].

Although the benefits of co-design have been discussed at length in the literature, there are few oral health examples of how this process has been used in the development of complex public health interventions [50] to generate collaborations and outcomes between researchers, service users and staff [30]. As described previously, the interaction between health visitors and families acts as a key ‘touchpoint’ [51] where value could be added in encouraging optimal oral health habits from an early age. However, the complexity of the PSB behaviour along with the need for an acceptable intervention required a pragmatic approach. The intervention had to be straightforward to use and easy to understand, whilst incorporating sufficient depth to address the complex behaviours and numerous barriers to developing and maintaining optimal home-based oral health behaviours and habits.

Various different approaches have been formulated, including Transdisciplinary Action Research [52–54], Co-production [30, 31, 55] or Experience-Based Co-Design [28]. The various methodologies for the co-design element of intervention development and the approach taken by the research team is often influenced by the time and funding available. Our approach was underpinned by our generic PSB intervention, which followed the MRC complex intervention framework guidance [39]. The adaptation of this generic model to an intervention delivered through a specific delivery vehicle allowed for the consideration of contextual factors. The staged approach taken had the benefits of consultation with both key and wider stakeholders, which allowed for a wide range of opinions to be considered and incorporated into the intervention from the start of the process. Early inclusion of the healthcare teams and parents in the development of the resources and how HABIT was delivered, e.g. the delivery protocol for the visit, enable consensus and “buy in” to be reached. This was fundamental to the development of an acceptable and feasible intervention.

The first stage of the process, gathering resources used by health visitors, allowed for key stakeholder input to explore both current practice and the views of other health promotion professionals. The workshops and interviews with parents and health visitors developed an understanding of their key priorities, which informed not only the content and format of the resources, but also the method of delivery. The peer review ensured that all messages were appropriate and aligned with current guidance and the dissemination element encouraged further feedback from not only those who had been directly involved in the study, but also wider stakeholders. The dissemination event was a key step in the process as it allowed for feedback, which will go on to inform the refinement stage of resource development. As a wider group of delegates were invited to the event, the varying priorities and perspectives contributed to the rich information gathered. Finally, the verification, review and reflection of resources, confirmed that the resources were not only providing appropriate evidence-based advice, but also addressed the barriers to behaviour change by targeting the TDF behavioural determinants.

Advantages of participatory research designs include increased likelihood of the intervention or service being both feasible and acceptable to the target audience [31, 32, 55]. Iterative approaches where small groups are consulted about the various iterations of the intervention design on multiple occasions may benefit from the group members feeling more engaged with the process and initial ideas may be reflected on and developed further. For pragmatic / logistical reasons (e.g., sessions held during health visitor team meetings / nursery group sessions) the HABIT intervention and resource development was more appropriate to carry out using separate focus groups with health visitors and families. It was also felt that conducting the sessions in separate small groups of health visitors or parents, allowed participants to speak openly about any issues or concerns they had.

Although HABIT is a universal intervention to be delivered to all children aged 9–12 months, it was developed and embedded for us in an area with high deprivation. During the intervention development, co-design was undertaken with communities at high-risk of dental decay. This ensured that key high risk behaviours would be addressed and ensured, through the principles of Proportionate Universalism [56], that the HABIT intervention would be suitable as a universal intervention at low dose but also appropriate at higher doses for families requiring additional support. As this intervention is delivered by health visitors who, by nature of their role, offer visits soon after birth, there would be very few children / families who would not have access to an intervention delivered in this way.

### Key learning points

The use of video resources can be extremely valuable to portray information or messages, however, the time and effort involved in the filming and editing of this type of resource must be factored into timelines. Further information about video resource development has been included here as it is an important, but not well discussed aspect of resource development and intervention co-design. Updating or altering video resources can be challenging and costly. As this paper has highlighted, co-design is an iterative process, which by definition, has many stages and amendments.

- The creation of a storyboard is a crucial step of the process as it guides the direction of the filming required. The storyboard outlines the content and order for the planned video, which directs the filming and footage required.
- Consent processes must be carefully considered at the start of the project, as re-consenting takes time and may not always be possible from all of those involved.
- The parents felt that one-to-one conversations about oral health would be beneficial and that the health visitor was a trusted person who was well placed to have these discussions as they had already built up a trusting relationship.
- Both parents and health visitors talked about how prioritisation of information is of great importance to ensure that the most appropriate advice is provided at the most suitable developmental stage for the child. Parents also requested key information to be provided in a quick to read format without too much detail.
- Conversations relating to paper verses digital resources were had both at the initial interviews / workshops and the dissemination event, with a general consensus that a variety of formats would be beneficial.
- Digital resources have the flexibility to be adapted for use with wider audiences, e.g., subtitles in various languages, however over reliance on digital resources can exclude some families within the community.
- The Covid-19 pandemic has highlighted issues of digital poverty in the local area with only 62% of pupils accessing online teaching [57]. These issues can be transposed across to digital health related resources with families unable to access these resources owing to the lack of a device, sufficient memory space on their device or credit for internet access.

### Further iterations

Having followed the staged approach to co-designed resource development, which culminated in the verification, review and reflection of the resources, the HABIT intervention now requires further iterations to allow for wider and more targeted use. In line with the MRC complex intervention development framework [22], prior to moving forward to an effectiveness trial, the intervention resources are being reviewed and refined. One key aspect for development is to ensure that the resources are accessible to communities where they are most needed, focussing on high risk and vulnerable groups. This will involve adaptation of current resources to make sure they are both accessible and appropriate for use with wider community groups, e.g. subtitles in various languages and increased use of pictures / illustrations rather than text. Alongside this adaption of accessibility of the resources, work will be undertaken to widen the scope of the current resources, so they contain advice and support which is suitable for use with babies and children from birth to two years, rather than specifically for the 9–12 month visit.

The co-design of the Health Visitor protocol for use during the 9–12 month visit was a great strength of this project as it helped to ensure the feasibility of the intervention. Work as part of stages six and seven, has incorporated further co-design with Health Visitors to enhance the HABIT intervention training and to iteratively develop appropriate and acceptable methods to monitor fidelity of intervention delivery.

## Conclusions

The co-designed HABIT intervention developed with an oral health focus provides a framework and learning for multiple healthcare settings. When considering the development of a co-designed intervention, a structured pragmatic approach to the process is essential to ensure that the intervention is evidence-based, acceptable and feasible for the required context. The iterative methodology allows for re-evaluation and essential amendments to be incorporated and reviewed by both key and wider stakeholders.

## Supplementary Information

**Additional file 1. **GUIDED– a guideline for reporting for intervention development studies.

**Additional file 2. **Parents focus group - topic guide.

**Additional file 3. **Health visitors focus group - topic guide.

**Additional file 4. **Intervention development using TIDieR (template for intervention description and replication) checklist [38].

**Additional file 5. **Resources mapped to the Theoretical Domains Framework (TDF) [41, 42] and Delivering Better Oral Health [8] guidance.

## Data Availability

The datasets used and analysed during the current study are available from the corresponding author on reasonable request.

## Abbreviations

HABIT: Health Visitors delivering Advice in Britain on Infant Toothbrushing
MECC: Making Every Contact Count
MRC: Medical Research Council
NIHR: National Institute for Health Research
NIHR ARC YH: NIHR Applied Research Collaborations Yorkshire and Humber
PHE: Public Health England
PSB: Parental Supervised Brushing
TDF: Theoretical Domains Framework
