# Supplementary material for: Co-design of an oral health intervention (HABIT) delivered by health visitors for parents of children aged 9–12 months
Source: BMC Public Health. 2022 Sep 24;22:1818. doi: 10.1186/s12889-022-14174-w (PMC9508763; doi:10.1186/s12889-022-14174-w)
Supplement: Supplementary file 2 — Additional file 2. Parents focus group - topic guide. [file 12889_2022_14174_MOESM2_ESM.docx]

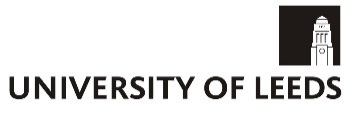
PARENTS FOCUS GROUP-TOPIC GUIDE

# Before the focus group starts:

- Specify how long the FG will take.
- Check that the 2 recorders have new batteries and are working properly before going into the FG.
- Bring extra copies of information sheets and consent forms.
- Make sure all consent forms are signed.
- Offer travel expenses form & stamped envelopes.
- Inform about the bathroom location

# Introduction:

- **Thank you for taking the time to attend this discussion and sharing with us your views and experiences regarding oral health advice you receive from Health Visitors and the resources they use in this process**
- **The purpose of this focus group is to increase our understanding of the situation and to co-produce and optimise the oral health advice provided by Health Visitors to parents at the universal home visit for infants aged 9-12 months**
- **We work as a team of experts in different fields and this FG and five other will help us refine our knowledge and develop questions for the second stage of the project where we will interview parents of infants aged 12 months, film them brushing babys’ teeth, measure plaque level and discuss what would help them in the process**
- **There are no right or wrong views and opinions and I am aware that this is an area with diverse views and practices.**
- **But first of all, let’s start going round the table introducing ourselves. Name, how many children do you have, how old are they, do you take care after them on your own or does somebody help you, do you work. In other words, a little bit of a backround, so we can get a better understanding.**
- **I’ll go first...**

# After the Focus Group:

- Thank the respondents
- Check if all resources are returned
- Remember to catalogue the tape/file
- During debriefing, reflect on, discuss with the research team and clearly note down any other issues (e.g. general impressions of the FG, questions that worked well, others that didn’t work) that may be important for future data collection, and data analysis
- Double check if everyone has reimbursement forms and envelops

Stage 1: Exploration of current toothbrushing practices

**20-30 min**

The questionnaire is founded on the refined Theoretical Domains Framework^[[1]](#footnote-1)^

| Exploration Areas | | Questions & Prompts |
| --- | --- | --- |
| **General practice of universal home visits** | | Have you already met a Health Visitor?   - If yes, how would you describe the experience? How was it? How would you describe a universal home visit? - If no, what do you know about Health Visitors and universal home visits? |
|  |  | Were/ are you able to choose your Health Visitor? |
|  |  | How often do/ will you see them? |
|  |  | If you already had a home visit, which topics did you discuss? Who chose them? |
|  |  | What is the most important issue or question you would like to find out regarding taking care after your baby? |
| **Current practice- Oral health advice** | | How important is infant’s oral health to you? |
|  |  | Would you like to learn more about infant’s oral health prevention and promotion? |
|  |  | What areas of oral health advice would you like the Health Visitor to address (toothbrushing, diet, others)? |
| **Framework Domain 1** | **Knowledge- Oral Health** | What do you know about oral health? |
|  |  | How familiar are you with how to take care of infant’s oral health? |
|  |  | Where do you get this knowledge from? |
|  |  | Is there anything you would like to find out or to learn about? |
|  | **Knowledge- Resources** | If you already had an interaction with a Health Visitor, did he/ she use any resources? What were they? |
|  |  | If you haven’t met a Health Visitor yet, have you heard anything about how they deliver the support and advice and what kind of resources they use? |
| **Framework Domain 2:** | **Skills- Oral Health** | We have talked a little bit about what you know about infant’s oral health and what about skills? Do you think you have enough skills to take care of infant’s oral health? Where did you learn this? |
|  |  | In your opinion, do other parents have enough skills to take care of infant’s oral health? |
|  | **Skills- Resources** | Would you like a Health Visitor to train you how to brush kid’s teeth and take care of his/ her oral health? |
|  | **Social/ Professional Role and Identity-**  **Oral Health** | Do you think it is a Health Visitor who should provide oral health advice? |
| **Framework Domain 3:** |  | What about personality traits of a Health Visitor? What kind of characteristics should he/ she have? |
|  | **Social/ Professional Role and Identity- Resources** | As we have already discussed, Health Visitors use different resources when they meet families. Is there anything you would feel uncomfortable with in terms of using resources? |
| **Framework Domain 4:** | **Beliefs about Capabilities-**  **Oral Health** | How confident do you fell regarding taking care of your baby’s oral health? |
|  |  | Is there anything you find difficult or hard? |
|  | **Beliefs about Capabilities- Resources** | Is there anything you find difficult or feel that some additional support, information or training would help you? What are these areas? |
| **Framework Domain 5:** | **Optimism- Oral Health** | Do you think it is a good idea to include oral health advice into universal home visit package for families of infants 9-12 months?  Why yes? Why no? |
|  | **Optimism- Resources** | In your opinion, what kind of resources should Health Visitors use in order to assist you and other parents and to improve infant oral health? |
| **Framework Domain 6:** | **Beliefs about Consequences- Oral Health** | How do you think what are potential outcomes of taking and not taking care of infant’s oral health? |
|  | **Beliefs about Consequences- Resources** | Do/would you use the resources provided by Health Visitors in your everyday brushing practices? |
|  |  | What kind of assistance, support or resources would do you need, if any? |
| **Framework Domain 7:** | **Reinforcement-Oral Health** | Would you follow the advice provided by Health Visitors? |
|  |  | What would you do to ensure that it becomes a part of your daily routine? |
|  | **Reinforcement- Resources** | Would you purposefully seek to ensure you use the resources you provide by Health Visitors? How do you ensure that? How do you know? |
| **Framework Domain 8:** | **Intentions- Oral Health** | If you could rank oral health prevention advice in the context of other things and issues that you care about or want to know about taking care of a baby, where would you put oral health advice? Why there? |
|  | **Intentions- Resources** | Domain 6&7. |
| **Framework Domain: 9** | **Goals- Oral Health** | How much of a goal is oral health in the context of everything that you care and need to do in order to ensure infant’s wellbeing? |
|  | **Goals- Resources** | Would you seek to use the resources or advice received from a Health Visitor every day or adjust it to life circumstances? |
| **Framework Domain: 10** | **Memory, Attention and Decision Process-**  **Oral Health** | Do you always remember to do things that would ensure that baby’s oral health is good? |
|  |  | Is it easy to remember oral health- related things? |
|  |  | Is there anything you do/ have that helps you to remember? |
|  | **Memory, Attention and Decision Process-**  **Resources** |  |
|  |  |  |
| **Framework Domain 11:** | **Environmental Context and Resources- Oral Health** | What about your family members? Do they care about oral health? Theirs and the infant’s? |
|  | **Environmental Context and Resources- Resources** | In your home setting, would you be the only one who takes care of baby’s oral health or is there anyone else, who would help you? |
|  |  | What kind of help and support would it be? |
| **Framework Domain 12:** | **Social Influences- Oral Health** | What about your friends and other mothers? What is their position regarding oral health promotion and prevention? |
|  |  | Do you discuss oral health advice related issues with other parents? What are the main similarities and differences between your and their position and experience, if any? |
|  |  | Have you ever felt pressured by other people or your GP to do anything related to the infant’s oral health? |
|  | **Social Influences- Resources** | Do other HV in your organisation use the resources available? What is their position regarding them? |
|  |  | Do you discuss resources with other health visitors? |
|  |  | Are there any differences in the way resources are used by different professionals? |
| **Framework Domain 13:** | **Emotions- Oral Health** | How would you describe your feelings when you received the first oral health advice from a Health Visitor? |
|  | **Emotions- Resources** | How would you describe your feelings when using the resources a Health Visitor provided to you? |
|  |  | How do you feel using these resources on your own? |
| **Framework domain14:** | **Behavioural Regulation- Oral Health** | What is your typical preparation for brushing infant’s teeth or taking care after his/ her diet? |
|  |  | Do you need to do/ plan anything in advance? |
|  | **Behavioural Regulation- Resources** | What about the resources? Do you need to do any preparation or planning before you use them? |
|  |  | Do you have an opportunity to reflect on your experience of using resources or received advice and change or alter your everyday routine? |

Stage 2: Exploration of oral health promotion resources

Thank you very much for sharing your personal perspectives and experience about taking care of infant’s oral health. We already made some references to as well as briefly discussed the fact that some Health Visitors use different resources when they visit families. Now we would like to share the resources used by Health Visitors in different parts of the UK and find out what you think about them. This is what we have collected (share numbered resources). Please take a few minutes to familiarise with the materials, explore, check and try them. Then select four resources that you think you would find useful when taking care after infant’s oral health and fill in the ‘If you want to achieve Y in situation S, something like X might help’ form.

## Discussion on the four choices (**10-15 min**)

- Could you please introduce the group with your choice of the 4 resources?
- Why did you choose these and not other resources?
- Would you use these materials in practice?
- If yes, how? If no, why?
- Would you know how to use them?
- What are their weaknesses?
- What improvements are needed?

Thank you very much! What a fascinating discussion.

## Pair discussion (**5-10 min**)

Now we would like to ask you to work in pairs. Identically to the activity when you had to select 4 resources, we now would like to ask you to select and agree on 3 resources in total as a pair. Please discuss, negotiate and as a pair select 3 resources that both of you would find the most useful or important in everyday practice. Agree and define key touch points where improvements might be made or service improved. If you think that we have missed anything and both of you think that there is something else that we have not presented to you, but you would use it in practice, please take the prepared sheet of paper and let us know about it.

## Concluding group discussion (**15-20min**)

Now, could each group present the choice of 3?

- Why did you decide to choose resources and not others? What is so special about them?
- Would you feel confident in using these resources when providing support?
- Are there any structural/ institutional/ administrative barriers that may prevent you from using these resources?
- What are you key messages regarding oral health interventions provided to parents and use materials and resources in the process?
- Is there anything else you would like to say that we have not asked?

## End

Thank you for your participation. We appreciate your time and a possibility to learn from your experience and expertise.

Go back to the check-list on the front page.

1. # James Cane, Denise O’Connor and Susan Michie (2012). Validation of the theoretical domains framework for use in behaviour change and implementation research. *Implementation Science,* **7**:37

   [↑](#footnote-ref-1)
