# Supplementary material for: Co-design of an oral health intervention (HABIT) delivered by health visitors for parents of children aged 9–12 months
Source: BMC Public Health. 2022 Sep 24;22:1818. doi: 10.1186/s12889-022-14174-w (PMC9508763; doi:10.1186/s12889-022-14174-w)
Supplement: Supplementary file 3 — Additional file 3. Health visitors focus group - topic guide. [file 12889_2022_14174_MOESM3_ESM.docx]

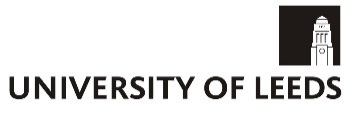
HEALTH VISITORS FOCUS GROUP-TOPIC GUIDE

# Before the focus group starts:

- Specify how long the FG will take.
- Check that the 2 recorders have new batteries and are working properly before going into the FG.
- Bring extra copies of information sheets and consent forms.
- Make sure all consent forms are signed.
- Offer travel expenses form & stamped envelopes.
- Inform about bathroom location

# Introduction:

- **Thank you for taking the time to attend this discussion and sharing with us your views and experiences regarding oral health advice you provide to parents of infants aged 9-12 months and the resources you use in this process**
- **The purpose of this focus group is to increase our understanding of the situation and to co-produce and optimise the oral health advice provided by Health Visitors to parents at the universal home visit for infants aged 9-12 months**
- **We work as a team of experts in different fields and this FG and five other will help us refine our knowledge and develop questions for the second stage of the second stage of the project where we will interview parents of infants aged 12 months, film them brushing infants’ teeth, measure plaque level and discuss what would help them in the process**
- **There are no right or wrong views and opinions and I am aware that this is an area with diverse views and practices.**
- **But first of all, let’s start going round the table introducing ourselves. Name, where do you work and perhaps you would like to say why you decided to take part in this focus group today.**
- **I’ll go first...**

# After the Focus Group:

- Thank the respondents
- Remember to catalogue the tape/file
- During debriefing, reflect on, discuss with the research team and clearly note down any other issues (e.g. general impressions of the FG, questions that worked well, others that didn’t work) that may be important for future data collection, and data analysis

Stage 1: Exploration of current professional practices

**20-30 min**

The questionnaire is founded on the refined Theoretical Domains Framework^[[1]](#footnote-1)^

| Exploration Areas | | Questions & Prompts |
| --- | --- | --- |
| **General practice of universal home visits** | | Could you provide us with a profile of a typical family to whom you provide a universal home visit? Is there such thing as a typical family? |
|  |  | How do these families get ‘ascribed’ to you? |
|  |  | How often do you see the families? |
|  |  | How does the home visit look like? |
|  |  | Who decides which topics should be addressed: parents or you? |
|  |  | What is the most specific/ unique thing when delivering a universal home visit to parents of infants 9-12 months aged? |
| **Current practice- Oral health advice** | | Do you address infant’s oral health in the intervention? |
|  |  | How does it look like? |
|  |  | What areas of oral health advice does it concentrate on (toothbrushing, diet, others)? |
| **Framework Domain 1** | **Knowledge- Oral Health** | What do you know about oral health? |
|  |  | How familiar are you with preventative oral health advice and support for families of infants aged 9-12 months? |
|  |  | In your opinion, what are the main strengths of what you know? |
|  |  | What about the weaknesses? |
|  |  | In your opinion, do you and other Health Visitors have enough knowledge about the subject? |
|  |  | Where do you get this knowledge from? |
|  |  | What do you know about what parents know? What do they know? |
|  | **Knowledge- Resources** | Do you use any resources when advising families on oral health? |
|  |  | How do you feel using them? |
|  |  | How do you know how to use them? |
|  |  | How do parents and infants respond to the materials you use regarding oral health and care? |
| **Framework Domain 2:** | **Skills- Oral Health** | And what about the skills? Do you think you have skills to provide preventative oral health support? |
|  |  | In your opinion, do other HV have enough skills to provide the service? |
|  |  | In an ideal world, what kind of skills should the HV have to provide comprehensive, acceptable and quality oral health advice to families? |
|  | **Skills- Resources** | And what about skills for using the resources you already have? What kind of skills are needed, if any? |
|  |  | Do you think you have these skills? |
|  |  | Did you receive any training on how to use the resources? Was it helpful? What was the most/ least helpful? |
|  | **Social/ Professional Role and Identity-**  **Oral Health** | Do you think it is HV who should provide oral health advice? |
| **Framework Domain 3:** |  | To what extent is providing this kind of support and advice part of your responsibility? |
|  |  | What about you as a person in this role? Have ever experienced any tensions between your personal position/ feelings/ identity and the professional role to provide oral health support for families of infants? |
|  | **Social/ Professional Role and Identity- Resources** | What is your professional opinion about the resources you use? Are they compatible with your professional responsibilities? |
|  |  | Do you feel comfortable using them or should other professionals, like dentists, be involved in this kind of support? Why? |
| **Framework Domain 4:** | **Beliefs about Capabilities-**  **Oral Health** | How confident do you fell in providing oral health advice in general and to this infant-age group of parents, in specific? |
|  |  | Is there anything you find difficult or hard to advice? |
|  | **Beliefs about Capabilities-**  **Resources** | How confident and comfortable do you feel using the resources you have? |
|  |  | Is there anything you find difficult or feel that some additional support, information or training would help you? What are these areas? |
| **Framework Domain 5:** | **Optimism- Oral Health** | Do you think it is a good idea to include oral health advice into universal home visit package for families of infants 9-12 months? Why yes? Why no? |
|  | **Optimism- Resources** | Do you think using the resources you use or could use has a potential to assist families and improve infant oral health? |
| **Framework Domain 6:** | **Beliefs about Consequences- Oral Health** | How do you think what are potential outcomes of the advice and support you provide? |
|  | **Beliefs about Consequences- Resources** | How do you think the resources you use impact the final outcome? |
|  |  | Do you think parents find the resources helpful and adopt them in their everyday brushing practices? |
| **Framework Domain 7:** | **Reinforcement-Oral Health** | Do you seek to ensure that families follow your provided advice? How do you ensure that? |
|  |  | Does the carrot or a stick is a better means to make sure families take care of infant’s oral health? |
|  | **Reinforcement- Resources** | Do you seek to make ensure families use the resources you provide them? How do you ensure that? How do you know? |
| **Framework Domain 8:** | **Intentions- Oral Health** | If you could rank oral health prevention advice in the context of other things and issues that you discuss with parents of infants aged 9-12 months, where would you put oral health advice? |
|  |  | Do you address infant oral health advice in each universal home visit, or does it vary? What are the factors for differences? |
|  | **Intentions- Resources** | Do you intend to use resources in every home visit, or does it vary on the situation? What are the factors for the differences? |
| **Framework Domain: 9** | **Goals- Oral Health** | How much of a goal is oral health in the context of everything you do in universal home visits? |
|  |  | How much do you want to improve infant oral health? |
|  |  | When talking about infant’s oral health, what is your main goal? |
|  | **Goals- Resources** | Do you seek to use the resources you have in every home visit or it depends. If it depends, what are the factors? |
|  |  | What is your main goal in using the resources? |
| **Framework Domain: 10** | **Memory, Attention and Decision Process-**  **Oral Health** | Do you always remember to provide oral health advice? |
|  |  | Is it easy to remember to talk about oral health? |
|  |  | How do you make sure you do not forget to address this issue? |
|  | **Memory, Attention and Decision Process-**  **Resources** | Do you always remember to use the resources when talking about oral health? |
|  |  | Is it easy to forget some of them? What do you do to make sure you do not forget? |
| **Framework Domain 11:** | **Environmental Context and Resources- Oral Health** | Do you get enough support from your organisation regarding oral health promotion and prevention? What is that support or training? |
|  |  | Do you feel your organisation provides you with needed skills and knowledge needed to support parents on their infants’ oral health? |
|  |  | What is your organisation’s position on oral health? How strongly it is incorporated in the overall agenda? |
|  | **Environmental Context and Resources- Resources** | What about the resources? What do you get? Is it enough? Does the organisation take into account your preferences for or knowledge about needed resources? |
|  |  | How do you get the resources from your organisation? |
| **Framework Domain 12:** | **Social Influences- Oral Health** | What about other HV in your organisation? What is their position regarding oral health promotion and prevention? Do they deliver this kind of advice and support? |
|  |  | Do you discuss oral health advice related issues with other health visitors? What are the main similarities and differences between your and their position and experience, if any? |
|  |  | Have you ever felt pressured by your colleagues or the organistion to provide or to not provide oral health advice? |
|  | **Social Influences- Resources** | Do other HV in your organisation use the resources available? What is their position regarding them? |
|  |  | Do you discuss resources with other health visitors? |
|  |  | Are there any differences in the way resources are used by different professionals? |
| **Framework Domain 13:** | **Emotions- Oral Health** | How would you describe your feelings when delivering advice on oral health promotion and prevention? |
|  |  | Do you like talking with parents about oral health? |
|  | **Emotions- Resources** | How would you describe your feelings when using the resources you have? |
|  |  | Are there any circumstances when you experience unusual or uncommon feelings or emotions when using the resources? What are these circumstances? |
| **Framework domain14:** | **Behavioural Regulation- Oral Health** | What is your typical preparation for a universal home visit and in particular oral health advice provision? |
|  |  | Do you need to do/ plan anything in advance? |
|  | **Behavioural Regulation- Resources** | What about the resources? Do you need to do any preparation or planning before you use them? |
|  |  | Have an opportunity to reflect on your experience of using resources and change or alter the practice? |

Stage 2: Exploration of oral health promotion resources

Thank you very much for sharing your own professional experience of using different materials for oral health interventions. Now we would like to look at broader practices and resources available nationally. You will be presented with a set of the PSB techniques, resources and procedures that are used by your colleagues across the UK. Please take few minutes to familiarise with the materials, explore, check and try them. Then each of you will be asked to reflect on the materials.

## General overview (**10-15 min**)

- Would you use these materials in practice?
- If yes, how? If no, why?
- In your opinion, what are their strengths?
- What are their weaknesses?
- What improvements are needed?

Thank you very much! Now each of you please select four resources that you would find useful in your everyday professional practice when assisting parents to adopt PSB techniques and generic skills needed to ensure infant’s oral health. After choosing the 4 resources, please fill in the ‘If you want to achieve Y in situation S, something like X might help’ form. The answers provided in the form do not need to be related to your selected resources. Try to think in a broader context of your practices and situations that you deal with in universal home visits.

## Discussion on the four choices (**10-15 min**)

- Could you please introduce the group with your choice of the 4 resources?
- Why did you choose these and not other resources?
- Would you use these materials in practice?
- If yes, how? If no, why?
- Would you know how to use them?
- What are their weaknesses?
- What improvements are needed?

Thank you very much! What a fascinating discussion.

## Pair discussion (**5-10 min**)

Now we would like to ask you to work in pairs. Identically to the activity when you had to select 4 resources, we now would like to ask you to select and agree on 3 resources as a pair. Please discuss, negotiate and as a pair select 3 resources that both of you find the most useful or important in everyday professional practice. Agree and define key touch points where improvements might be made or service improved. If you think that we have missed anything and both of you think that there is something else that we have not presented to you, but you would use it in your practice, please take the prepared spread sheet and let us know about it.

## Concluding group discussion (**15-20min**)

Now, could each group present the choice of 3?

- Why did you decide to choose resources and not others? What is so special about them?
- Would you feel confident in using these resources when providing support?
- Are there any structural/ institutional/ administrative barriers that may prevent you from using these resources?
- What are you key messages regarding oral health interventions provided to parents and use materials and resources in the process?
- Is there anything else you would like to say that we have not asked?

## End

Thank you for your participation. We appreciate your time and a possibility to learn from your experience and expertise.

Go back to the check-list on the front page.

1. # James Cane, Denise O’Connor and Susan Michie (2012). Validation of the theoretical domains framework for use in behaviour change and implementation research. *Implementation Science,* **7**:37

   [↑](#footnote-ref-1)
