# Supplementary material for: Co-design of an oral health intervention (HABIT) delivered by health visitors for parents of children aged 9–12 months
Source: BMC Public Health. 2022 Sep 24;22:1818. doi: 10.1186/s12889-022-14174-w (PMC9508763; doi:10.1186/s12889-022-14174-w)
Supplement: Supplementary file 5 — Additional file 5. Resources mapped to the Theoretical Domains Framework (TDF) [41, 42] and Delivering Better Oral Health [8] guidance. [file 12889_2022_14174_MOESM5_ESM.docx]

Additional File 5: Resources mapped to the Theoretical Domains Framework (TDF) (39, 40) and Delivering Better Oral Health (8) guidance.
